# Study on the Electrochemical Reaction Mechanism of ZnFe_2_O_4_ by *In Situ* Transmission Electron Microscopy

**DOI:** 10.1038/srep28197

**Published:** 2016-06-16

**Authors:** Qingmei Su, Shixin Wang, Libing Yao, Haojie Li, Gaohui Du, Huiqun Ye, Yunzhang Fang

**Affiliations:** 1 Zhejiang Provincial Key Laboratory of Solid State Optoelectronic Devices, Zhejiang Normal University, Jinhua, 321004, China; 2 Institute of Physical Chemistry, Zhejiang Normal University, Jinhua, 321004, China

## Abstract

A family of mixed transition–metal oxides (MTMOs) has great potential for applications as anodes for lithium ion batteries (LIBs). However, the reaction mechanism of MTMOs anodes during lithiation/delithiation is remain unclear. Here, the lithiation/delithiation processes of ZnFe_2_O_4_ nanoparticles are observed dynamically using *in situ* transmission electron microscopy (TEM). Our results suggest that during the first lithiation process the ZnFe_2_O_4_ nanoparticles undergo a conversion process and generate a composite structure of 1–3 nm Fe and Zn nanograins within Li_2_O matrix. During the delithiation process, volume contraction and the conversion of Zn and Fe take place with the disappearance of Li_2_O, followed by the complete conversion to Fe_2_O_3_ and ZnO not the original phase ZnFe_2_O_4_. The following cycles are dominated by the full reversible phase conversion between Zn, Fe and ZnO, Fe_2_O_3_. The Fe valence evolution during cycles evidenced by electron energy–loss spectroscopy (EELS) techniques also exhibit the reversible conversion between Fe and Fe_2_O_3_ after the first lithiation, agreeing well with the *in situ* TEM results. Such *in situ* TEM observations provide valuable phenomenological insights into electrochemical reaction of MTMOs, which may help to optimize the composition of anode materials for further improved electrochemical performance.

The ever–growing need for high energy density, power density and stable cyclability has prompted considerable attention to develop promising anode materials for lithium ion batteries (LIBs) to meet the rapid development of portable electronics. Various transition metal oxides (TMOs), such as iron oxides^1^,^2^,^3^, cobalt oxides^4^,^5^ manganese oxides^6^,^7^ and nickel oxides^8^,^9^, have been competitive anode candidates for LIBs due to their stable capability and high reversible capacities (500–1000 mAh g^−1^)^8^,^10^,^11^. Recently, iron oxides anode has received an upsurge of interest due to their fascinating superiority. However, the poor electrical conductivity, severe volume expansion and higher oxidation potential restrict their applications on LIBs^12^. Furthermore, the rapid capacity fading of iron oxides anode materials remains a major drawback. In particular, the Fe_2_O_3_ anode materials have been found to be irreversible in the first lithiation by *in situ* transmission electron microscopy (TEM); they undergo a reversible phase conversion between FeO and Fe/Li_2_O during the lithiation-delithiation cycles^13^. The Fe_2_O_3_ anode materials cannot recover to their original structure after delithiation, which cause a large capacity loss in the first cycle. Recently, Fe–based oxides (AFe_2_O_4_, A = Mn^14^, Co^15^, Ni^16^, Cu^17^ and Zn^18^, etc) have been studied as promising anodes for LIBs to improve their cyclability. It is anticipated that they can effectively overcome the drawbacks of pure iron oxide anode; then larger reversible capacity, better cyclability, and better rate performance can be achieved by the suitable combination of different metal species^19^.

ZnFe_2_O_4_ stands out from other Fe-based spinel series anodes because of its high abundance and high theoretical specific capacity of 1072 mAh g^−1^ 20. Qin *et al*. first reported the use of ZnFe_2_O_4_ as an anode synthesized by a pulsed laser deposition method^21^, and their initial reversible capacity was 556 mAh g^−1^ and after100 cycles 78% of the capacity (434 mAh g^−1^) was still retained. Then the electrochemical performances of diverse ZnFe_2_O_4_ nanostructure have been greatly enhanced, such as ZnFe_2_O_4_/C hollow spheres^22^ ZnFe_2_O_4_ octahedrons^23^ ZnFe_2_O_4_ nanofibers (ZFO–NF) and nanorods^20^, and so on. However, a few of the fundamental mechanism concerning the electrochemical process remain unclear. Wang’s group^24^ have reported that the products of the deeply discharged are LiZn and Fe, and the recharged materials are ZnO and Fe_2_O_3_, which is distinguishing from the previous *in situ* TEM study on Fe_2_O_3_ anode^13^. While, Chowdari B.V.R. *et al*.^25^ have suggested that the reaction mechanism of ZnFe_2_O_4_ is reversible reactions of LiZn to ZnO and Fe to FeO after the first discharge process. Particularly, the dynamic electrochemical reaction of binary transition metal oxide for LIBs is still in a black box.

*In situ* TEM technique has been recognized as an excellent option to monitor real–time observation of electrode materials with lithium and sodium on the nanometer scale^26^,^27^,^28^,^29^. Some successes have been achieved on understanding the electrochemical mechanism of SnO_2_^30^, Si^31^,^32^, ZnO^33^, CeO_2_^34^, Fe_2_O_3_^13^, carbon nanotube (CNT)^35^, graphene^36^, and Co_9_S_8_/CNT^37^ in real time through the *in situ* TEM technique. Up to now, the electrochemical reaction between ZnFe_2_O_4_ and Li has not been studied. Here, an all–solid nano–LIB was constructed inside a high–resolution TEM using ZnFe_2_O_4_ as working electrode to visualize the microstructure and phase evolution during electrochemical processes. It is found that upon lithiation the ZnFe_2_O_4_ nanoparticle was converted into numerous Fe and Zn nanograins within Li_2_O matrix with a severe volume expansion. During delithiation, the anode cannot be converted to its original phase ZnFe_2_O_4_ but transformed to Fe_2_O_3_ and ZnO. The Fe valence evolution of ZnFe_2_O_4_ nanoparticle is also studied by EELS measurements, which agrees well with the *in situ* TEM results. Our *in situ* TEM results for provided the direct experimental evidence of the reaction mechanism of ZnFe_2_O_4_ during lithium-ion insertion and extraction.

## Results

**Figure 1:**
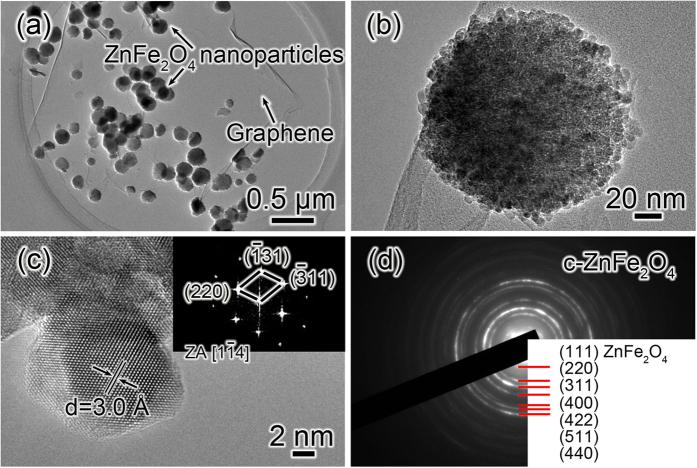
(**a**) TEM, (**b**) The high–magnification TEM, (**c**) HRTEM, and (**d**) ED pattern of the obtained products ZnFe_2_O_4_/graphene electrode.

The microstructure characterization of the obtained ZnFe_2_O_4_/graphene is shown in Fig. 1. Figure 1(a) is the TEM image of ZnFe_2_O_4_/graphene, it indicates there are many ZnFe_2_O_4_ particles with sizes of 120~180 nm anchored on graphene, and the transparent nature of the graphene implies that it is fully exfoliated into single or few-layer sheet. A high–magnification TEM image of an individual ZnFe_2_O_4_ nanoparticle is given in Fig. 1(b). Obviously, the ZnFe_2_O_4_ nanoparticle is primarily composed of nanocrystals with a size ~10 nm. The smaller size of ZnFe_2_O_4_ nanocrystals can shorten Li^+^ diffusion pathways, increase the electron/ion conductance, and reduce the volume change induced by lithiation/delithiation, further enable the ZnFe_2_O_4_ nanocrystals to show an improved electrochemical performance. The high resolution transmission electron micrograph (HRTEM) was taken along the 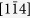 zone axis with the (220) lattice finger directly seen with a spacing of 0.30 nm as shown in Fig. 1(c); the corresponding fast Fourier transform (FFT) is shown in the inset of Fig. 1(c), in accordance with the (220), 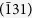, and (311) planes of the cubic structure of ZnFe_2_O_4_ (JCPDS card no. 89–1012). Figure 1(d) presents an ED pattern recorded from the synthesized ZnFe_2_O_4_/graphene. All the diffraction rings can be perfectly indexed as a cubic structure of ZnFe_2_O_4_ (JCPDS card no. 89–1012); it further confirms that the resultant products are ZnFe_2_O_4_ phase.

**Figure 2:**
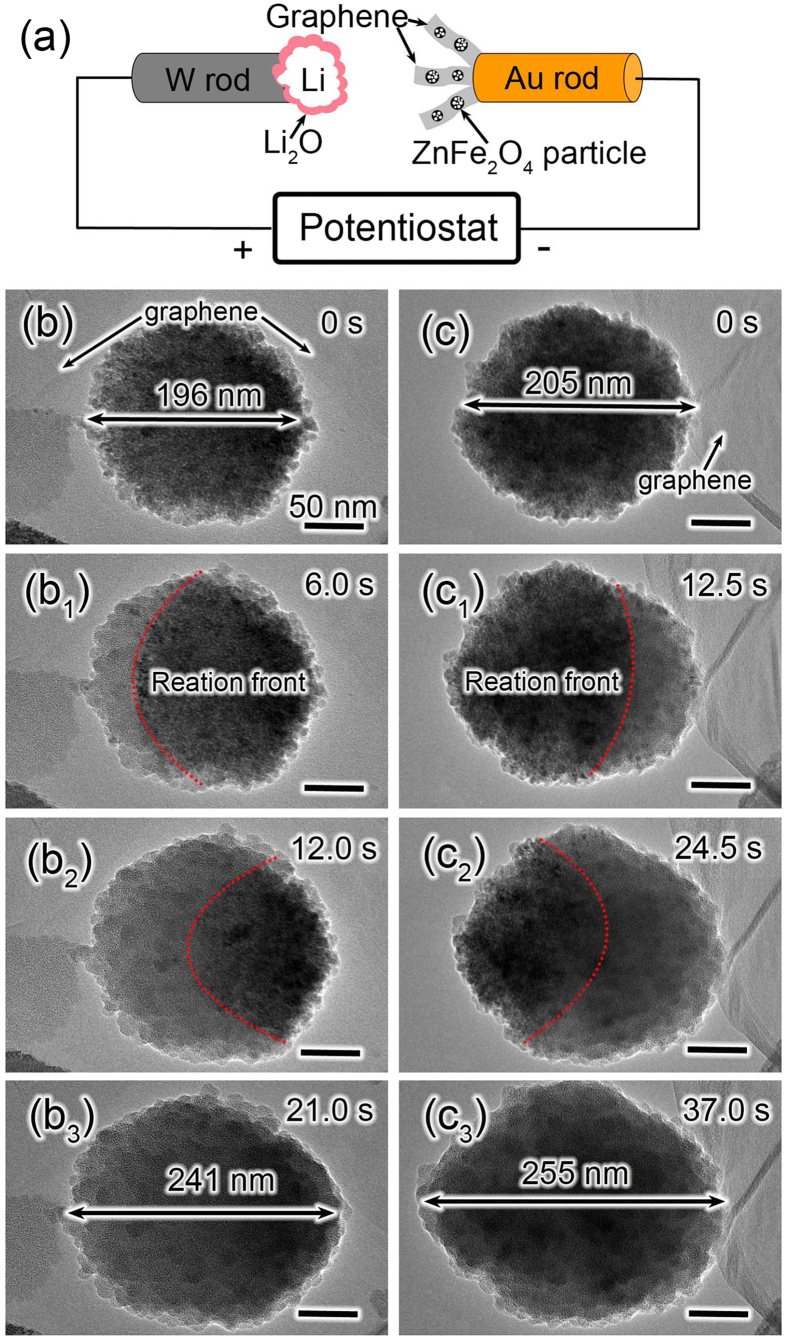
Morphological changes of two ZnFe_2_O_4_ particles during lithiation with a potential of −1 V. (**a**) Schematic illustration of the *in situ* nano-battery setup. (**b–b**_**3**_) TEM snapshots show the morphological evolution of a ZnFe_2_O_4_ particle anchored on graphene with a diameter of ~196 nm. (**c–c**_**3**_) TEM snapshots show the morphological evolution of a second ZnFe_2_O_4_ particle sited on the edge of graphene with a diameter of ~205 nm. The red dashed curves demonstrate the reaction front. All the scale bars are 50 nm.

To investigate the electrochemical behavior of ZnFe_2_O_4_ during lithiation–delithiation cycles, an *in situ* nanoscale electrochemical device of ZnFe_2_O_4_ was constructed, as schematically shown in Fig. 2(a). Briefly, the electrochemical nano–LIB device consists of three essential components: ZnFe_2_O_4_/graphene anode, metal Li counter electrode, and the naturally grown solid electrolyte Li_2_O layer on metal Li. After contact between Li_2_O and ZnFe_2_O_4_/graphene anode was established, a constant potential of −1.0 V was applied to the ZnFe_2_O_4_/graphene against to the Li counter electrode to drive the first lithiation of ZnFe_2_O_4_. Figure 2(b,c) and Supplementary Movie S1,S2 show the morphological changes of two ZnFe_2_O_4_ nanoparticles with diameters of ~196 and 205 nm in the first electrochemical lithiation process. It suggests that the lithiation occurred on all surfaces within 37.0 s, indicating the fast lithium diffusion on the ZnFe_2_O_4_ nanoparticle surface. As shown in Fig. 2(b), a ~23% size increase from 196 to 241 nm in the diameter was seen for the ZnFe_2_O_4_ nanoparticle anchored on graphene layer, and the first lithiation lasted for 21 s, giving a lithiation ratio of ~2.14 nm/s for this ZnFe_2_O_4_ nanoparticle. As for another ZnFe_2_O_4_ nanoparticle sited on the edge of the graphene (Fig. 2c), its diameter expanded from 205 nm to about 255 nm after full lithiation within 37.0 s, and the lithiation ratio of this ZnFe_2_O_4_ nanoparticle is about 1.25 nm/s, which is much smaller than that of ZnFe_2_O_4_ nanoparticle on graphene layer. This can be attributed to the good Li^+^ conductivity of graphene. In other words, the good Li^+^ conductivity of graphene sheets makes the fast diffusion of lithium around ZnFe_2_O_4_ nanoparticle.

**Figure 3:**
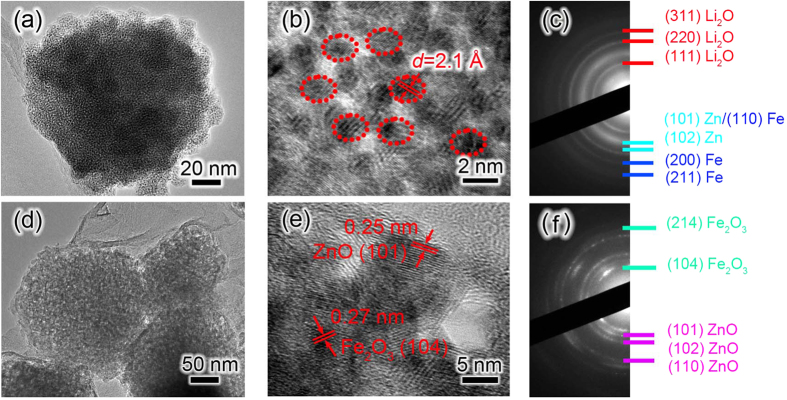
(**a**) TEM and, (**b**) HRTEM images of an individual lithiated ZnFe_2_O_4_ particle, (**c**) ED pattern of the lithiated ZnFe_2_O_4_/graphene electrode, (**d**) TEM and (**e**) HRTEM images of delithiated ZnFe_2_O_4_ particle, (**f**) ED pattern of the delithiated ZnFe_2_O_4_/graphene electrode.

Close view on the microstructure of ZnFe_2_O_4_ nanoparticle after full lithiation and delithiation is shown in Fig. 3. Figure 3a,b is the TEM image of an individual ZnFe_2_O_4_ nanoparticle anchored on graphene after the first lithiation. It shows that many small nanograins with sizes of 1–3 nm dispersed in Li_2_O matrix, saying after the first lithiation process Fe and Zn nanocrystals embedded in Li_2_O matrix uniformly, as evidenced by HRTEM and ED in Fig. 3b,c. From the HRTEM image of the fully lithiated ZnFe_2_O_4_ nanoparticle that displayed in Fig. 3b, the lattice spacing recorded from the nanocrystals is about 2.10 Å, agrees with the (110) plane of Fe and (101) plane of Zn. Figure 3c is the ED pattern collected from the lithiated ZnFe_2_O_4_/graphene electrode. The diffraction rings corresponding to the (111), (220), (311) lattice planes of Li_2_O are marked by red lines, while the (110), (101) lattice planes of Fe and Zn have been labeled. Then the potential was reversed to + 3 V to initiate the delithiation process, and the TEM images of the electrode after full delithiation are shown in Fig. 3d,e. When the electrode was fully delithiated, the clear lattice fringes (0.25 and 0.27 nm) correspond to (101) plane of phase ZnO and (104) plane of phase Fe_2_O_3_ respectively. Also the ED pattern (Fig. 3f) confirms the presence of Fe_2_O_3_ and ZnO in the delithiated product, rather than ZnFe_2_O_4_. As for ZnFe_2_O_4_ electrode, in the most favorable case, the Fe^2+^ ions in FeO would be further reversibly oxidized to form trivalent Fe^3+^ ions to obtain Fe_2_O_3_ due to the synergetic effects of Zn and Fe in ZnFe_2_O_4_ electrode^23^,^24^. In addition, no Li_2_O diffraction ring was detected from the ED pattern, suggesting that the Li_2_O was almost consumed up during delithiation process.

**Figure 4:**
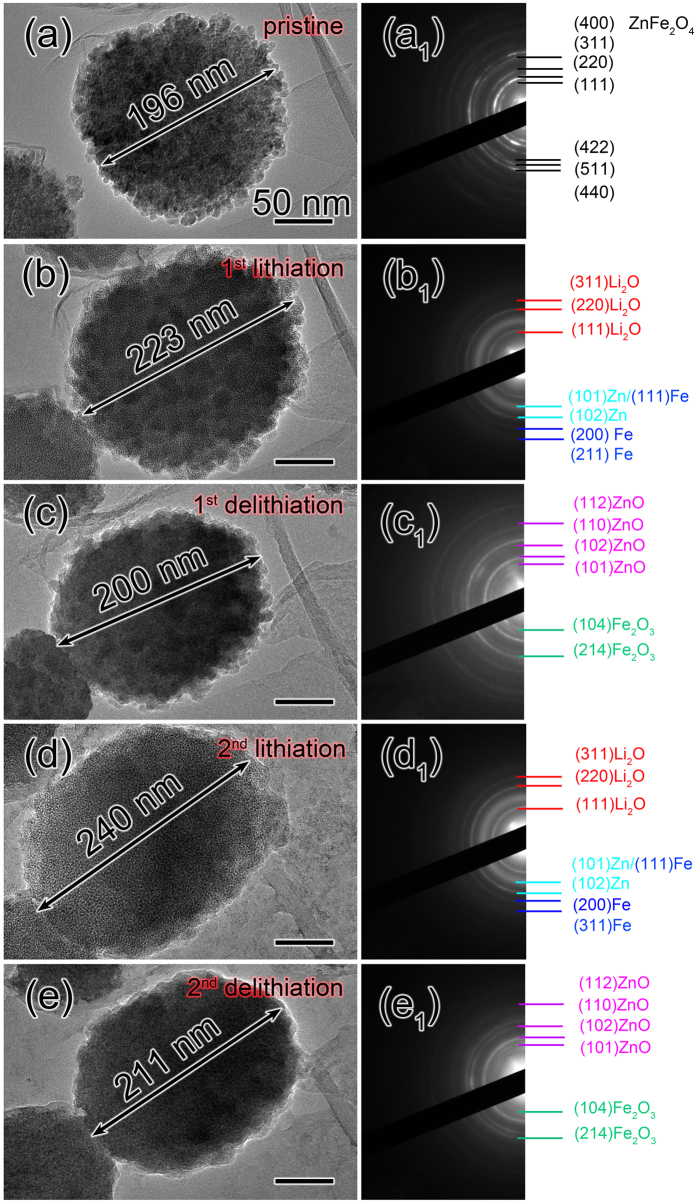
Morphological and microstructural changes of ZnFe_2_O_4_/graphene electrode during the first two lithiation-delithiation cycles. (**a–e**) Morphological changes of ZnFe_2_O_4_ particle in the first two lithiation-delithiation cycles. (**a**_**1**_–**e**_**1**_) ED patterns recorded from the corresponding ZnFe_2_O_4_/graphene electrode in (**a–e**).

We next concern about the electrochemical behaviors of ZnFe_2_O_4_/graphene electrode during lithiation/delithiation cycles. A ZnFe_2_O_4_ particle with a nearly spherical shape and the initial diameter of ~196 nm is selected to check the morphology evolution, as shown in Fig. 4a. The ED pattern of the obtained ZnFe_2_O_4_/graphene electrode is given in Fig. 4a1. It can be perfectly indexed as the face–centered crystal structure of ZnFe_2_O_4_ (JCPDS no. 89–1012). The pristine ZnFe_2_O_4_ nanoparticle was inflated and expanded its size to 223 nm after full lithiation (Fig. 4b). The ED pattern of the fully lithiated ZnFe_2_O_4_/graphene electrode is shown in Fig. 4b1; the diffraction rings can be well indexed as Fe, Zn and fcc Li_2_O, suggesting the ZnFe_2_O_4_ was transformed to Fe, Zn and fcc Li_2_O after the first full lithiation process. Then the potential was reversed to + 3 V to facilitate the delithiation process. Along with the first delithiation process, volume contraction observed throughout the whole nanosphere with the size decreasing from 223 nm to 200 nm. The fully delithiated phase was identified as Fe_2_O_3_ and ZnO, as examined by the ED pattern of the delithiated ZnFe_2_O_4_/graphene electrode. Then the second lithiation/delithiation cycle was investigated by reversing the applied potential of −1 and + 3 V, as shown in Fig. 4c–e. The TEM image of ZnFe_2_O_4_ particle after the second lithiation process is given in Fig. 4c; the marked ZnFe_2_O_4_ particle expanded its size to ~240 nm again, just as the first lithiation process. The ED pattern that recorded from the lithiated ZnFe_2_O_4_/graphene electrode after the second lithiation is shown in Fig. 4d1, it indicates the product was Fe, Zn and cubic Li_2_O. The lithiated ZnFe_2_O_4_ particle shrunk its size to 211 nm again in the second delithiation process showing the reversible micromorphology change, as displayed in Fig. 4e. Figure 4e1 shows the corresponding ED pattern of the delithiated ZnFe_2_O_4_/graphene electrode, and the ED pattern confirms the resultant Fe and Zn nanograins transformed to Fe_2_O_3_ and ZnO again in the second delithiation process. As discovered above, all the TEM results indicate the reversible conversion from Fe to Fe_2_O_3_ and Zn to ZnO after the first lithiation process.

**Figure 5:**
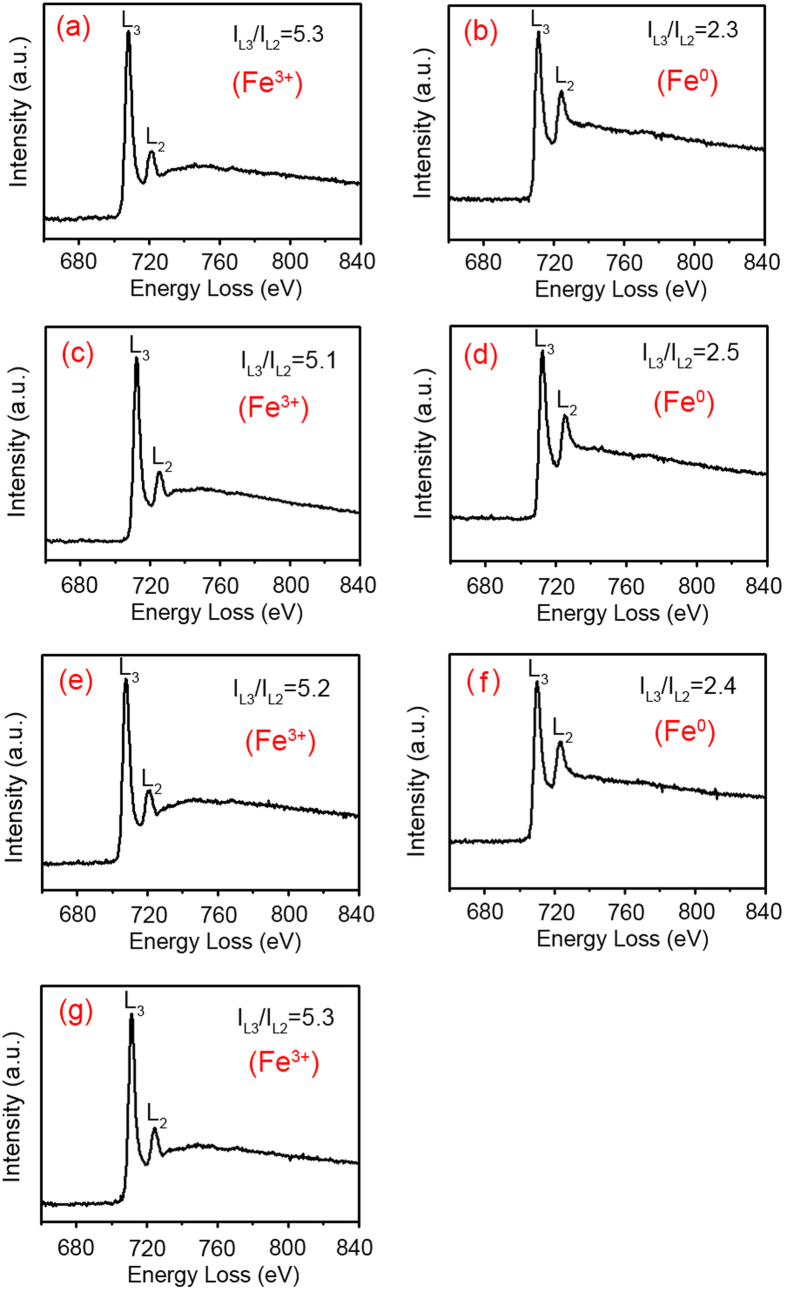
EELS spectra of iron from ZnFe_2_O_4_ nanoparticle during the lithiation and delithiation cyclings. (**a**) The initial stage, (**b**) the first lithiated state and (**c**) the first delithiated state, (**d**) after the second lithiation and (**e**) delithiation process, and (**f**) after the third lithiation and (**g**) delithiation process. The periodic fluctuation of white–line intensity ratio (L_3_/L_2_) of Fe in the lithiation/delithiation cycle implies the reversible transformation of Fe^0^ and Fe^3+^.

An EELS that assisted in TEM is a useful technique for analyzing the valences of some transition metal elements at the nanoscale. The transition of an electron from a 2p level to 3d orbitals leads to the formation of L_2,3_ white lines due to the unoccupied 3d orbitals of transition metals^38^. The L_3_/L_2_ white−line intensity ratio (I_L3_/I_L2_) measured in 3d transition metal is used to determine the occupation number of 3d electrons. Here the I_L3_/I_L2_ of Fe has been obtained to correlate EELS features with the valence states of Fe in the first three lithiated and delithiated states. Figure 5 shows the EELS results of Fe collected from the lithiated and delithiated ZnFe_2_O_4_/graphene electrode to confirm the evolution of valence states of Fe elements in the electrochemical lithiation and delithiation cycles. The EELS spectrum of Fe in the original ZnFe_2_O_4_/graphene electrode is shown in Fig. 5a, we can see that the L_3_/L_2_ intensity ratio of Fe is 5.3, confirming that the valence state of Fe is undoubtedly 3 + 39. The L_3_/L_2_ intensity ratio of Fe in the fully lithiated stage reduced to 2.3, which is smaller than that of Fe^2+^ (4.1 ± 0.2), agrees well with the valence state of 0^40^, as given in Fig. 5b, suggesting the oxide state transition of Fe from 3+ to zero. From the EELS spectrum of Fe recorded from a fully delithiated ZnFe_2_O_4_/graphene anode that shown in Fig. 5c, in which the L_3_/L_2_ intensity ratio of Fe increased to 5.1, this EELS can be regarded approximately complete oxidation, that is, Fe “3 +” fingerprint. This result confirms that after the first cycle the delithiated product was Fe_2_O_3_. It says the Fe element can renew its original state of Fe^3+^ after the first delithiation process, further demonstrating good reversibility of Fe metal. Figure 5d displays the L_3_/L_2_ intensity ratio of Fe is 2.5 after the second full lithiation process, which is similar with that of Fe in the first lithiated stage, corresponding to the valance state of zero. Then the L_3_/L_2_ intensity ratio of Fe increased to 5.2 again calculated from the EELS spectrum given in Fig. 5e, it implies that the valance of Fe element is +3 after the second delithiation process. Expectedly, the similar reversal of L_3_/L_2_ intensity ratio of Fe in ZnFe_2_O_4_ was also noticed in the third cycle. The repeated changes in L_3_/L_2_ intensity ratio of Fe indicate the complete and reversible electrochemical transition between Fe^0^ and Fe^3+^ during the electrochemical processes, thus leading to high reversibility for iron oxide based anodes. The EELS results are agree well with the ED results shown in Fig. 4a1–e1, which reveal that the electrochemical reaction of iron oxide phase in ZnFe_2_O_4_ during the electrochemical processes is a reversible phase transition between Fe and Fe_2_O_3_ after the first lithiation process.

**Figure 6:**
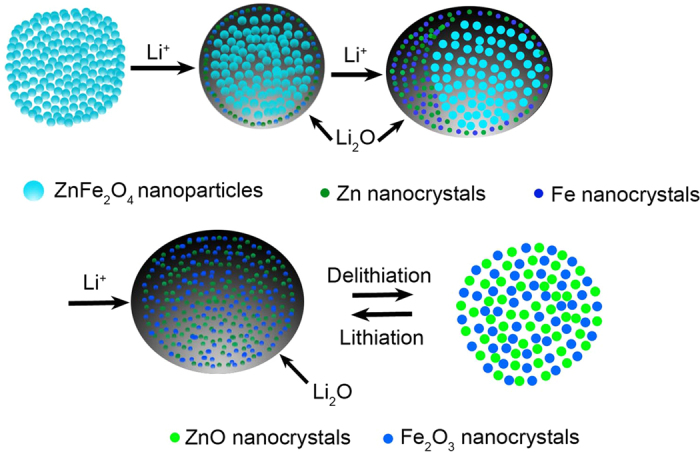
Schematic illustration of the conversion reaction of an individual ZnFe_2_O_4_ nanoparticle.

According to the above *in situ* TEM analysis, the possible reactions of ZnFe_2_O_4_ during the lithiation/delithiation process are proposed as follows. During the first lithiation process, Li^+^ ions can diffuse quickly on graphene sheets, leading to a uniform lithiation take place on the surface of ZnFe_2_O_4_ nanoparticles. As Li^+^ insertion continued, the lithiation front in the ZnFe_2_O_4_ gradually propagated and gave rise to a visible interface between the lithiated and unlithiated phases. As a result, lithiation is essentially the destruction of the crystal structure, lithium is intercalated into ZnFe_2_O_4_ and led to metallic Zn, Fe nanograins and Li_2_O appear in the product followed by the obvious volume expansion. The first lithiation can be expressed as: ZnFe_2_O_4_ + 8Li^+^ +  8e^−^ → Zn + 2Fe + 4Li_2_O. During the delithiation process, Li-ion will firstly be extracted from the lithiated ZnFe_2_O_4_ leading to volume contraction, and lithiated product metallic Fe and Zn nanograins can be oxidized to metal oxide (Fe_2_O_3_ and ZnO) with the presence of Li_2_O through the conversion reaction, ZnFe_2_O_4_ is not the initial molecule that can be recovered. So the delithiation process can be described as: Zn + Li_2_O → ZnO + 2Li^+^ + 2e^−^, 2Fe + 3Li_2_O → Fe_2_O_3_ + 6Li^+^ + 6e^−^. In the following lithiation/delithiation cycles, the reversible conversion reaction of ZnO, Fe_2_O_3_ and metallic Zn, Fe nanoparticles take place and indicates good reversibility. Thus, after the first cycle, reversible reactions can be expressed as following equations: ZnO + 2Li^+^ + 2e^−^
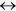
Zn + Li_2_O, Fe_2_O_3_ + 6Li^+^ + 6e^−^
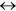
2Fe + 3Li_2_O. Figure 6 schematically outlines these changes of the ZnFe_2_O_4_ nanoparticles during lithiation/delithiation cycles.

Therefore, the stable cycling response of ZnFe_2_O_4_ (see Supplementary Fig. S1) may be ascribed to not only the synergistic effect of different type metal oxide species (Zn and Fe) on ZnFe_2_O_4_, but also a facile and easier lithium ion diffusion on graphene during the lithiation/delithiation cycles. Also, in the following lithiation/delithiation cycles, ZnO and Fe_2_O_3_ convey reversible electrochemical reactivity toward Li and then reveal a reversible phase conversion of Zn-ZnO and Fe-Fe_2_O_3_, accounting for good reversibility and high Coulombic efficiency.

## Conclusions

In summary, the electrochemical reaction mechanism of ZnFe_2_O_4_ for lithium ion battery anode is investigated by *in situ* TEM, and the results show that in the first lithiation process lithium-ion is intercalated into ZnFe_2_O_4_, generating ultrafine (1–3 nm) Fe and Zn nanocrystallites within Li_2_O matrix followed by obvious volume expansion. In the first delithiation process, the HRTEM and ED results show that ZnFe_2_O_4_ is not the original molecule that can be recovered, but metallic Zn and Fe nanoparticles oxidized to their respective metal oxides ZnO and Fe_2_O_3_ with the disappearance of Li_2_O through the complete conversion reaction. The ED patterns and EELS spectra reveal that the electrochemical lithiation/delithiation processes of ZnFe_2_O_4_ nanoparticles as anode in LIBs are revealed to be reversible phase transition between Fe, Zn nanograins and Fe_2_O_3_, ZnO nanograins. The information obtained from our findings can help to further improve the electrochemical performance of this type material and also is insightful for exploring various types of electrode materials in LIB technology.

## Methods

### Materials synthesis

Graphite oxide (GO) was synthesized by a modified Hummers’ method^41^. ZnFe_2_O_4_/graphene was prepared by a hydrothermal route. Firstly, GO (100 mg) was dispersed in ethylene glycol (80 ml) with sonication for 30 min to form a homogeneous dispersion. Then, Zn(Ac)_2_ H_2_O (0.55 g), FeCl_3_ (0.81 g), and NaAc (3.6 g) were added into the above solution with stirring for 30 min, and the mixture was transferred into a Teflon–lined autoclave with a capacity of 100 mL, and maintained at 200 °C for 24 h. The precipitate was isolated by filtration and washed several times after cooling down to room temperature. Finally, the product was obtained by drying the precipitate at 60 °C for 12 h.

### *In situ* TEM electrochemical setup

The nano-LIBs experimental set-up was constructed inside a TEM (JEOL JEM–2100F) to enable the *in situ* observation on the electrochemical behaviors of ZnFe_2_O_4_ anode with the assistance of HRTEM, electron diffraction (ED) and EELS measurements. The ZnFe_2_O_4_ nanoparticles anchored on graphene were used as the working electrode, and Li metal was coated onto a piezo–driven W probe and regarded as lithium source and counter electrode, an oxide layer of Li_2_O formed on Li metal when it is exposed to air act as the solid electrolyte. The detailed setup procedure for the nano-LIB can be found in literature^13^. When ZnFe_2_O_4_/graphene driven by the nanomanipulator of the TEM-STM holder is contact with the Li_2_O layer, a nano-LIBs cell is successfully constructed. After that, a potential of −1 V was applied to the ZnFe_2_O_4_ electrode against the Li source to drive Li^+^ transport to initiate lithiation process, and then the bias was reversed to + 3 V to facilitate delithiation. EELS measurements were performed on a TEM with the assistance of Gatan EELS attachments.

## Additional Information

**How to cite this article**: Su, Q. *et al*. Study on the Electrochemical Reaction Mechanism of ZnFe_2_O_4_ by *In Situ* Transmission Electron Microscopy. *Sci. Rep.* **6**, 28197; doi: 10.1038/srep28197 (2016).

## Supplementary Material

Supplementary Information

Supplementary Information

Supplementary Information
