# Supplementary material for: Study on the Electrochemical Reaction Mechanism of ZnFe2O4 by In Situ Transmission Electron Microscopy
Source: Sci Rep. 2016 Jun 16;6:28197. doi: 10.1038/srep28197 (PMC4910063; doi:10.1038/srep28197)
Supplement: Supplementary Information [file srep28197-s1.docx]

**Study on the Electrochemical Reaction Mechanism of ZnFe_2_O_4_ by *In Situ* Transmission Electron Microscopy**

Qingmei Su^1,*^, Shixin Wang^1^, Libing Yao^2^, Haojie Li^2^, Gaohui Du^2,*^, Huiqun Ye^1^, &Yunzhang Fang^1^

^1^ Zhejiang Provincial Key Laboratory of Solid State Optoelectronic Devices, Zhejiang Normal University, Jinhua, 321004, China

^2^ Institute of Physical Chemistry, Zhejiang Normal University, Jinhua, 321004, China

*Correspondence and requests for materials should be addressed to Q.S. (email: [suqingmei@zjnu.cn](mailto:suqingmei@zjnu.cn)) or G.D. (email: [gaohuidu@zjnu.edu.cn](mailto:gaohuidu@zjnu.edu.cn))

**1. Experimental Details**

**Half Cell Measurement.** Galvanostatic charge/discharge cycle was performed with a 2032–type coin cell between 0.01 and 3 V at room temperature on a Neware battery cycler at a current density of 100 mA g^−1^. To prepare the LIB electrode, the slurry was made by mixing active material, acetylene black, and polyvinylidene fluoride (PVDF) in a weight ratio of 75:15:10 in N–methyl pyrrolidine with stirring for 2 h. The slurry was coated onto Ni foam current collector and dried at 120 °C under vacuum overnight. Metal Li was used as the counter electrode in the test cells, which also functioned as the reference electrode. The electrolyte was 1.0 M LiPF_6_ in a mixture of ethylene carbonate (EC)/dimethyl carbonate (DMC) (1:1 volume ratio).

**2. Supporting Movies**

Movie_S1.mov

*In situ* TEM movie shows the electrochemical lithiation process of ZnFe_2_O_4_ particle anchored on graphene. The video was recorded at 4 frames/s.

Movie_S2.mov

*In situ* TEM movie shows the electrochemical lithiation process of the second ZnFe_2_O_4_ particle sited on the edge of graphene. The video was recorded at 4 frames/s.

**3. Supporting Figure**

The electrochemical performance of the ZnFe_2_O_4_/graphene composite as anode in LIBs has been measured at a current density of 100 mA g^-1^. As illustrated in Figure S1, the ZnFe_2_O_4_/graphene shows a specific capacity of ~1303 and 992 mAh g^-1^ for the first discharge and charge process, with a Coulombic efficiency of 76.1%. Its reversible capacity decreases gradually in the first 15 cycles and stabilizes at 870 mAh g^-1^ after 50 cycles.


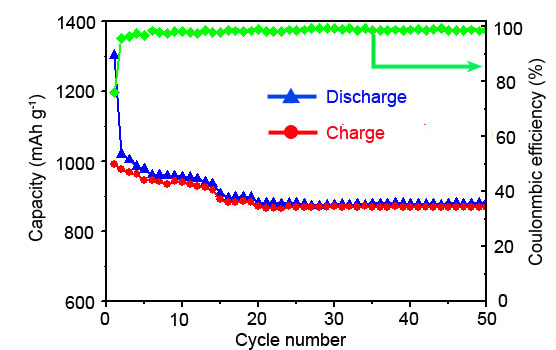


Figure S1. Specific capacity as a function of cycle number tested in a half cell configuration at a current density of 100 mA g^-1^.
